# Optimal Transport‐Based Transcriptomic Mapping Revealed Atypical Disease Progression Subtypes in Living Alzheimer’s Disease Patients

**DOI:** 10.1002/alz.092754

**Published:** 2025-01-03

**Authors:** Xiaoqing Huang, Jie Zhang, Kun Huang, Cristian A Lasagna Reeves, Nur Jury

**Affiliations:** ^1^ Indiana University, School of Medicine, Carmel, IN USA; ^2^ Department of Medical and Molecular Genetics, Indiana University, School of Medicine, Indianapolis, IN USA; ^3^ Indiana University, School of Medicine, Indianapolis, IN USA; ^4^ Stark Neurosciences Research Institute, Indiana University School of Medicine, Indianapolis, IN USA; ^5^ Department of Anatomy, Cell Biology, and Physiology, Indiana University School of Medicine, Indianapolis, IN USA

## Abstract

**Background:**

Alzheimer’s disease (AD) exhibits substantial heterogeneity in its disease trajectory. A subset of AD patients with unmatched cognitive decline/tauopathy severity has not been well studied. We identified such atypical subgroups in post‐mortem AD brain studies. However, such atypical subtypes may not be easily identified in living patients, as obtaining brain samples are unfeasible, and NFT measurement is not accurate. In this study, we utilize the matched transcriptomic data from both brain and blood of ROSMAP cohort to identify such atypical AD groups in the blood transcriptomic data of live patients in other cohorts using transfer learning‐based approach, to uncover distinct molecular signatures and biomarkers for earlier and more accurate disease subtyping and prognosis in living AD patients.

**Method:**

Three subgroups were defined from ROSMAP cohort with the blood and brain RNA‐seq data based on the clinical information of their tauopathies and disease progression, namely, Asymptomatic AD, Low‐NFT AD, Typical AD, plus normal Control, which serves as our training dataset for a supervised transfer learning. Then, the labels were transferred to the blood RNA‐seq samples from two new cohorts, ADNI and ANMerge using optimal transport. Next, we identify the genes consistently expressed in three independent cohorts for that specific AD subtype. Lastly, the diffusion pseudo‐time analysis infers the temporal order of the gene expression patterns within each subgroups. Dominant genes with a consistent expression pattern across cohorts are considered as the signature for each subgroup, and their relevance to AD pathology is analyzed.

**Result:**

We identified distinctive genes with consistent expression patterns across cohorts for each AD subgroup. Remarkably, our analysis also reveals the temporal gene expression dynamics differs for sex, age (late/early onset), and onset pattern (sudden/gradual) across the cohorts.

**Conclusion:**

Through a deep transfer learning‐based approach on the blood and brain transcriptomic data, we successfully identified the atypical disease progression subgroups among live AD patient cohorts in ADNI and ANMerge with promising biomarkers/gene signatures. The molecular signatures identified in this study not only enhance our comprehension of the underlying pathophysiological mechanisms but also hold promise for developing early prognosis and effective personalized treatments for AD and related tauopathies.

